# Diagnostic Challenges and Treatment Strategies in Neutrophilic Cicatricial Alopecias: A Narrative Review from Conventional Therapies to New Therapeutic Targets

**DOI:** 10.3390/life16050835

**Published:** 2026-05-19

**Authors:** Francesca Svara, Giulio Bortone, Luca Ambrosio, Felice Forte, Luca Gargano, Annunziata Dattola, Steven Paul Nisticò, Giovanni Pellacani, Carmen Cantisani

**Affiliations:** 1 Dermatology Unit, Department of Medical and Cardiovascular Sciences, “Sapienza” University of Rome, 00161 Rome, Italy; francescasvara@gmail.com (F.S.); giuliobortone93@gmail.com (G.B.); l.ambrosio@idi.it (L.A.); ff.feliceforte@outlook.it (F.F.); lucagargano1995@gmail.com (L.G.); nancydattola@gmail.com (A.D.); steven.nistico@uniroma1.it (S.P.N.); giovanni.pellacani@uniroma1.it (G.P.); 2 Istituto Dermopatico dell’Immacolata (IDI-IRCCS), Dermatological Research Hospital, 00167 Rome, Italy

**Keywords:** folliculitis decalvans, dissecting cellulitis of the scalp, cicatricial alopecia, neutrophilic primary cicatricial alopecias, systemic therapy, biologic treatments

## Abstract

Folliculitis decalvans (FD) and dissecting cellulitis of the scalp (DCS) are neutrophilic primary cicatricial alopecias characterized by chronic inflammation and irreversible hair loss, with distinct pathogenic mechanisms that make accurate diagnosis essential for appropriate management. This narrative review aims to provide a comprehensive overview of systemic therapeutic options for FD and DCS, to evaluate their efficacy in relation to underlying disease mechanisms, and to explore emerging targeted treatments. A literature search was conducted in PubMed/MEDLINE using relevant keywords related to neutrophilic cicatricial alopecias and therapeutic strategies, including studies reporting clinical outcomes in FD and DCS. Available evidence indicates that conventional therapies remain the cornerstone of management: antibiotics are typically first-line in FD, while isotretinoin represents the mainstay of treatment in DCS and a key option in refractory FD; however, these approaches are often associated with partial responses and frequent relapses. Biologic agents, particularly TNF-α inhibitors, have shown consistent benefit in refractory cases, while IL-17/23 and JAK inhibitors are supported by limited but emerging data. Overall, treatment response appears to reflect underlying pathogenic differences between FD and DCS, underscoring the importance of a tailored, mechanism-based approach. Further studies are needed to establish standardized treatment algorithms and confirm long-term efficacy and safety.

## 1. Introduction

Alopecia encompasses a heterogeneous group of disorders characterized by partial or complete hair loss and is broadly classified into non-scarring and scarring forms [1]. Primary cicatricial alopecias (PCA) represent a subset of inflammatory conditions in which the hair follicle is the primary target of destruction, ultimately leading to irreversible hair loss due to fibrotic replacement [2]. According to the North American Hair Research Society (NAHRS) classification, PCA are subdivided based on the predominant inflammatory infiltrate into lymphocytic, neutrophilic, mixed, and nonspecific forms [3]. Neutrophilic PCA include folliculitis decalvans (FD) and dissecting cellulitis of the scalp (DCS), which together account for approximately 20% of PCA cases [4]. However, this classification should be interpreted as a practical clinicopathological framework rather than as evidence of a purely neutrophilic infiltrate. Both FD and DCS may exhibit mixed inflammatory patterns that vary according to disease stage and biopsy site. In FD, early active lesions are typically characterized by neutrophil-predominant perifollicular inflammation, particularly involving the follicular infundibulum [5]. Increasing evidence suggests that Staphylococcus aureus plays a central role in the early phases of disease especially, likely in follicular structural abnormalities, microbiome dysbiosis, biofilm formation, and local immune dysregulation [6]. This initial neutrophilic inflammation may promote follicular destruction and immune privilege collapse, leading to secondary lymphocytic/lichenoid inflammation. Accordingly, advanced FD lesions often show a mixed inflammatory infiltrate with neutrophils, lymphocytes, plasma cells, histiocytes/macrophages, and foreign-body giant cells associated with fibrosis [5]. Similarly, DCS may show mixed neutrophilic, lymphocytic, and histiocytic inflammation even in relatively early stages, while chronic or advanced lesions are characterized by deep abscesses, sinus tract formation, granulomatous inflammation, foreign-body reaction, and scarring fibrosis [7,8]. Unlike FD, DCS belongs to the follicular occlusion spectrum and appears to be primarily driven by follicular hyperkeratosis and occlusion, leading to follicular rupture and subsequent deep dermal inflammation [7]. Bacterial colonization may occur in DCS lesions, particularly in advanced suppurative disease; however, their role is generally considered secondary rather than a primary pathogenic driver [1]. Thus, although FD and DCS are traditionally grouped within neutrophilic PCA, both disorders demonstrate a more complex and dynamic histopathological profile reflecting distinct pathogenic mechanisms and stage-dependent inflammatory changes. Clinically, FD presents with follicular pustules, crusting, and tufted hairs, typically affecting the vertex scalp, whereas DCS is characterized by inflammatory nodules, abscesses, and interconnected sinus tracts [8]. Beyond the risk of permanent hair loss, FD and DCS have a significant impact on patients’ quality of life. Chronic pain, recurrent inflammation, and visible scalp changes may lead to psychological distress, social withdrawal, and reduced self-esteem. These conditions often remain under-recognized and easily misdiagnosed, as they can overlap with other scarring and non-scarring alopecias [9]. Accurate differentiation is essential to avoid inappropriate treatments [10]. Therapeutic management remains challenging due to the absence of standardized treatment algorithms and the high rate of relapse. Among topical therapies, high-potency topical corticosteroids and intralesional corticosteroids represent first-line options for both FD and DCS, often combined with topical antibiotics in FD [11]. In moderate-to-severe forms, where topical therapy alone is insufficient to control disease activity, it remains useful as an adjunct to systemic treatment, contributing to local inflammation control and symptom management [12]. Given the substantial differences in underlying pathogenic mechanisms, FD and DCS should not be approached as a single therapeutic entity. Rather, optimal management requires a phenotype-driven strategy, in which treatment selection is guided by the relative contribution of microbial factors, follicular occlusion, and immune dysregulation. The aim of this review is to provide a comprehensive overview of systemic therapeutic options for neutrophilic PCA, with a particular focus on FD and DCS, and to critically evaluate their efficacy in relation to underlying pathogenic mechanisms. In addition, this work emphasizes the pivotal role of accurate diagnosis in guiding therapeutic decision-making and optimizing treatment selection and clinical outcomes. Finally, it explores emerging therapeutic strategies that may shape future approaches to the long-term management of these chronic conditions [13,14].

## 2. Materials and Methods

To provide a comprehensive overview of current evidence, we performed a literature review using the PubMed/MEDLINE database (National Library of Medicine, Bethesda, MD, USA). The literature search covered publications from January 2015 through March 2026, and the PubMed search was last updated in March 2026. The search strategy combined relevant keywords, including “primary cicatricial alopecia”, “folliculitis decalvans”, “dissecting cellulitis of the scalp”, and “neutrophilic scarring alopecia”, along with treatment-related terms such as “therapy”, “management”, “biologic therapy”, and “JAK inhibitors”, using Boolean operators (“AND”, “OR”). Only articles in English were included. Duplicate records were removed, and titles and abstracts were screened for relevance. We included prospective and retrospective cohort studies, case series, case reports, clinical trials, and systematic reviews. Articles not reporting therapeutic outcomes or not specifically addressing FD or DCS were excluded. Although this review is narrative in nature, an effort was made to include the most relevant and recent evidence available.

## 3. Results

### 3.1. Study Characteristics

A total of 46 articles met the inclusion criteria. The included studies were heterogeneous in design, comprising mainly retrospective cohorts, case series, and individual case reports, with only a limited number of prospective studies. Sample sizes varied considerably across studies, ranging from small case reports to multicentre retrospective cohorts. Most studies focused on treatment outcomes, with relatively few addressing long-term disease control or relapse rates. In addition, outcome measures were not standardized, and definitions of clinical response differed between studies, limiting direct comparability of results.

### 3.2. Folliculitis Decalvans

#### 3.2.1. Antibiotics

Antibiotics represented the most frequently reported therapeutic class and were commonly used as initial systemic treatment. Tetracyclines exert anti-inflammatory effects by inhibiting neutrophil chemotaxis and matrix metalloproteinase activity, thereby targeting the perifollicular inflammation characteristic of FD [15]. In clinical practice, their use is supported by retrospective data showing consistent, although often partial, responses. In the multicenter study by Vañó-Galván et al., tetracyclines were among the most used treatments and were associated with clinical improvement, although the duration of response was limited, highlighting their role in disease control rather than sustained remission [16]. In more severe or refractory cases, the clindamycin–rifampicin combination is the most consistently reported regimen. The most commonly used protocol consists of clindamycin 300 mg twice daily combined with rifampicin 300 mg twice daily for approximately 10–12 weeks. Its rationale lies in combined antimicrobial and anti-inflammatory activity, including biofilm penetration. In the same multicenter cohort by Vañó-Galván et al., this combination achieved high initial response rates, although relapse after discontinuation was frequent [16]. Similarly, the retrospective comparative study by Tietze et al. demonstrated that a substantial proportion of patients experienced recurrence shortly after treatment cessation [17]. These findings are reflected in EADV recommendations, which advise limiting repeated antibiotic courses [6]. Macrolides and trimethoprim–sulfamethoxazole have been reported less frequently, with available evidence largely limited to small series [18]. While some degree of clinical improvement has been described, these agents do not appear to significantly alter long-term disease course.

#### 3.2.2. Isotretinoin

Isotretinoin was reported across multiple retrospective cohorts, either as monotherapy or in combination with antibiotics, most often in patients with persistent or refractory disease. Although FD is not classically categorized within follicular occlusion disorders, isotretinoin may exert therapeutic effects through modulation of sebaceous activity, normalization of follicular keratinization, and attenuation of innate immune signaling. Clinical data support its use, particularly in refractory disease, showing remission durations ranging from 4 to 24 months [19]. In the retrospective study by Tietze et al., approximately 90% achieved sustained remission during treatment and for up to two years after discontinuation, compared with 33% in the clarithromycin group and 20% in those treated with rifampicin–clindamycin [17]. Similarly, Aksoy et al. conducted a retrospective study involving 39 patients, reporting an overall response rate of 82%, with 66% of patients remaining relapse-free when treated with doses of at least 0.4 mg/kg/day for a minimum of three months [20]. This observation is further supported by subsequent reviews and the EADV position statement, which highlight isotretinoin as a viable systemic option beyond traditional antibiotic approaches. Nevertheless, relapse after discontinuation remains common, and maintenance regimens have been described in several cohorts.

#### 3.2.3. Immunomodulatory Therapies

Hydroxychloroquine may be beneficial in selected cases with features suggestive of mixed inflammatory patterns and in rare phenotypic variants such as the folliculitis decalvans–lichen planopilaris spectrum, in which a shift toward a lymphocytic inflammatory profile may justify its use [21]. In a recent retrospective study, Doche et al. reported clinical improvement when hydroxychloroquine was used as adjunctive therapy in patients with persistent inflammatory activity [22]. Cyclosporine has also been reported as a potential therapeutic option in refractory FD, owing to its potent immunosuppressive effects on T-cell activation and cytokine production. Small case series have described clinical improvement, particularly in patients with prominent inflammatory activity, supporting its use mainly as a short-term rescue therapy or adjunctive agent in selected cases rather than as a long-term maintenance treatment. In the series by Jerjen et al., disease control was achieved in all treated patients; however, relapse occurred within 4–5 months after treatment discontinuation. Its long-term use is limited by the risk of significant adverse effects, including nephrotoxicity, hypertension, and increased susceptibility to infections [23]. Dapsone represents another mechanistically relevant option as a second line or adjunctive, due to its anti-neutrophilic effects [24]. A retrospective comparative study involving 28 patients evaluated the efficacy of different treatments for FD. Dapsone demonstrated moderate results, achieving stable remission in 43% of cases, which was higher than that observed with the clindamycin–rifampicin combination (20% remission) and clarithromycin (33%), but lower than isotretinoin, which achieved stable remission in 90% of patients [17].

#### 3.2.4. Adjunctive Therapies

Several adjunctive and procedural treatments have been explored in refractory FD, mainly as complementary strategies aimed at reducing inflammation and microbial burden. The beneficial effect of photodynamic therapy (PDT) is likely related to the generation of reactive oxygen species, leading to the modulation of local inflammatory pathways. In a retrospective series, Yang et al. reported significant clinical improvement and sustained disease control at 12 months in most patients [25,26]. Similarly, Collier et al. described prolonged remission following PDT in a recalcitrant case [27]. However, the heterogeneity of available data limits conclusions regarding long-term efficacy. Laser-based therapies, including long-pulsed Nd:YAG and diode lasers, have been investigated with the aim of reducing follicular inflammation and bacterial load. Preliminary reports suggest potential clinical benefit although evidence remains limited [28]. Platelet-rich plasma has been reported only in small case series. Its potential benefit may be related to the release of growth factors and cytokines that promote tissue repair and modulate local inflammatory responses. Suh et al. described symptomatic improvement in two refractory patients, although the small sample size precludes definitive conclusions [29].

#### 3.2.5. Biologic Therapies

Biologic escalation is increasingly considered in patients unresponsive to conventional antimicrobial and anti-inflammatory therapies [30]. TNF-α inhibitors, particularly adalimumab, are the most frequently reported agents [31,32,33]. Evidence from small case series and retrospective reports suggests clinical improvement, supporting a role for TNF-mediated pathways in sustaining chronic neutrophilic inflammation [34,35]. Notably, the relatively reproducible clinical responses observed across independent reports, together with the established safety profile of anti-TNF agents in other chronic inflammatory dermatoses, position this class as the most promising biologic strategy currently available for FD, particularly in severe and relapsing cases. The IL-23/Th17 axis represents an additional therapeutic target, given its involvement in neutrophil recruitment and amplification of inflammatory cascades [36]. Case reports, such as that by Ismail et al., have described successful treatment of refractory FD with secukinumab, providing proof-of-concept for IL-17 blockade [37]. However, evidence remains limited to isolated observations, and the heterogeneity of response suggests that Th17-driven inflammation may play a contributory rather than dominant role in all patients. JAK inhibitors offer broader suppression of cytokine signaling [11]. In a recent case series, Moussa et al. reported improvement in inflammatory signs and symptoms with baricitinib in patients with refractory FD, suggesting a potential role for targeting multiple inflammatory pathways simultaneously [38]. The rationale for JAK inhibition in FD is supported by the central role of the JAK–STAT pathway in mediating key pro-inflammatory cytokines involved in disease pathogenesis, including IL-1β and IL-8. Immunohistochemical studies have demonstrated selective overexpression of JAK3 and TYK2 in FD lesions, while their expression is absent in healthy controls. This pattern suggests a disease-specific activation of JAK signaling pathways. Consequently, both selective JAK3 inhibitors and pan-JAK inhibitors may represent promising targeted therapeutic strategies in refractory FD [39]. Despite this theoretical advantage, current data are preliminary, and long-term efficacy and safety in FD remain to be established. More recently, phosphodiesterase-4 inhibition has emerged as an additional therapeutic strategy [12]. Through modulation of intracellular cyclic AMP and downstream reduction in pro-inflammatory cytokines, Apremilast has shown clinical benefit in isolated case reports of refractory FD [40,41].

### 3.3. Dissecting Cellulitis of the Scalp

#### 3.3.1. Antibiotics

Antibiotics are frequently used in DCS, particularly during early inflammatory stages or in the presence of active suppuration and suspected secondary bacterial colonization [11]. Tetracyclines, especially doxycycline, are among the most used agents and may provide partial clinical improvement through inhibition of pro-inflammatory cytokine signaling. This makes them particularly useful in early disease phases characterized by inflammatory nodules and abscesses before extensive sinus tract formation has occurred [11]. However, available data consistently indicate that their efficacy is limited in chronic disease, where structural changes predominate. Similarly, rifampicin-based regimens, including rifampicin–clindamycin, as well as macrolides and other antibiotics, have been reported in multiple case series [12]. These treatments may reduce drainage, tenderness, and inflammatory lesion counts, particularly in patients with active suppuration. However, their effects are generally transient, and recurrence after treatment discontinuation is frequently observed.

#### 3.3.2. Isotretinoin

Isotretinoin represents the most consistently supported systemic therapy in DCS and directly targets the central pathogenic mechanism of follicular occlusion by normalizing follicular keratinization and decreasing hyperkeratotic plugging [42]. In addition, isotretinoin exerts anti-inflammatory actions that may contribute to reductions in deep dermal inflammation and suppurative lesions. Evidence from systematic reviews and pooled analyses indicates overall response rates of approximately 80–90%, with relapse occurring in approximately 20–25% of patients after treatment discontinuation [43]. Across studies, isotretinoin has been associated with reductions in nodules, abscesses, and drainage. Despite these favorable outcomes, relapse remains a clinically relevant issue, suggesting that isotretinoin suppresses disease activity without fully eliminating the underlying predisposition. This has led to the use of prolonged or maintenance regimens in selected patients [7].

#### 3.3.3. Biologic Therapies

Biologic therapies are increasingly used in moderate to severe or refractory DCS, particularly in patients with extensive inflammatory disease or overlap with hidradenitis suppurativa (HS), reflecting the shared pathogenic features within the follicular occlusion spectrum [44,45]. TNF-α inhibitors have the most robust evidence base in DCS, consistent with the central role of TNF-α in mediating neutrophil recruitment, chronic inflammation, and tissue destruction. Notably, adalimumab, currently approved for HS, has shown consistent efficacy in reducing inflammatory burden in follicular occlusion disorders. In a multicentre retrospective study, treatment with anti-TNF agents was associated with significant reductions in inflammatory nodules, abscess formation, and pain scores, along with improvements in quality of life [46]. The IL-17 and IL-23 pathways represent additional targeted approaches based on the role of the Th17 axis in neutrophil-driven inflammation. This is further supported by the demonstrated efficacy of IL-17 inhibitors, such as secukinumab and bimekizumab, in the treatment of HS [47]. In DCS, case reports have described clinical improvement with agents such as ixekizumab and tildrakizumab, particularly in patients with refractory disease [48,49]. These findings support a potential role for Th17-mediated pathways in sustaining inflammation, although evidence remains limited. JAK inhibitors have emerged as another promising therapeutic class. By inhibiting intracellular signaling of multiple cytokines, these agents provide broader immunomodulatory effects [50]. Case reports have described rapid reductions in inflammatory activity and improvement in symptoms [51,52]. A systematic review including 81 patients with DCS treated with immunomodulatory agents identified apremilast among the evaluated small-molecule inhibitors. Its use was associated with clinically meaningful improvement and a favorable safety profile; however, the current evidence remains limited and further studies are required to confirm its efficacy and long-term outcomes [49].

#### 3.3.4. Surgery

Surgery plays a more established and distinct role in DCS compared with FD, reflecting the structural nature of advanced disease [53]. In patients with localized disease characterized by established sinus tracts, abscess cavities, and extensive fibrosis, surgical excision can provide definitive removal of chronically diseased tissue. Case series have reported good outcomes following excision, particularly when combined with systemic therapy to control residual inflammatory activity and reduce recurrence risk [54]. In severe or extensive cases, surgical management may be considered, including staged excisions with healing by secondary intention. In very advanced disease, more aggressive approaches such as total scalpectomy followed by reconstruction with split-thickness skin grafts or free flaps, including latissimus dorsi flaps, may be required [53]. Additionally, incision and drainage remain useful for the management of acute abscesses [55]. However, surgery does not address the underlying inflammatory or occlusion-prone milieu, and recurrence in untreated areas remains possible. For this reason, a combined medical–surgical approach is often required in advanced disease.

## 4. Discussion

Neutrophilic PCA are chronic inflammatory disorders characterized by a predominantly neutrophilic or mixed neutrophilic–lymphocytic inflammatory infiltrate, ultimately leading to follicular destruction and irreversible scarring alopecia [4]. The importance of early and accurate diagnosis in FD and DCS is often underestimated. In fact, they are frequently misdiagnosed in their initial stages, often being confused with more common non-scarring conditions such as bacterial folliculitis, acneiform eruptions, seborrheic dermatitis, or even alopecia areata in early patchy presentations [11]. This diagnostic delay can result in inappropriate or insufficient treatments and may ultimately contribute to irreversible follicular damage and disease progression. In addition, both conditions must be distinguished from other forms of primary cicatricial alopecia; in particular, lichen planopilaris (LPP) and discoid lupus erythematosus (DLE). However, these conditions typically lack pustules and suppuration and show a lymphocyte-predominant infiltrate on histology [21]. Similarly, central centrifugal cicatricial alopecia (CCCA) may present with progressive scarring alopecia of the vertex but is usually not associated with prominent inflammation or pustulation [56]. Additionally, DCS should be differentiated from acne keloidalis nuchae (AKN), which may present with papules, pustules, and scarring localized to the occipital scalp and posterior neck. However, unlike DCS, AKN typically lacks deep abscesses and sinus tract formation and shows a more superficial, fibrosing inflammatory pattern [57]. The distinction between FD and DCS is also essential from a therapeutic standpoint, as their management strategies differ significantly. A multimodal diagnostic approach integrating clinical evaluation, dermoscopy, and histopathology is crucial to achieve an accurate diagnosis and guide appropriate management. Trichoscopy is a useful non-invasive tool that supports the clinical evaluation of FD and DCS, providing characteristic but not always pathognomonic features. In FD, trichoscopic findings typically reflect perifollicular inflammation and follicular damage, with hair tufting representing the most distinctive feature. This is often accompanied by perifollicular erythema, scaling or hair casts, pustules, yellow crusts, and erythematous erosions, predominantly at the periphery of active lesions [58]. In addition, mixed trichoscopic patterns with overlapping lymphocytic features, including prominent perifollicular scaling and erythema, may be observed in the FD–lichen planopilaris phenotypic spectrum [58,59]. In DCS, trichoscopy more commonly shows features related to deeper follicular destruction, including yellow structureless areas, dystrophic hairs, follicular pustules, keratotic plugs, yellow crusts, and follicular dropout, sometimes associated with signs of sinus tract formation and boggy inflammatory plaques [55] (see Table 1). When clinical and trichoscopic findings are inconclusive, histopathological examination remains essential for definitive diagnosis. In FD, histology typically shows inflammation centered on the upper portion of the hair follicle, with follicular destruction, fusion of follicular units, and progressive fibrosis; the inflammatory infiltrate may be mixed rather than purely neutrophilic [60]. In contrast, DCS is characterized by deeper dermal involvement, with abscess formation, follicular rupture, and the development of sinus tracts, often associated with a mixed suppurative and granulomatous inflammatory infiltrate [61]. Both conditions are typically chronic and relapsing, and prolonged treatment is often required to maintain disease control. As forms of scarring alopecia, the primary therapeutic objective is not hair regrowth but suppression of inflammation and prevention of further follicular destruction [8]. A stepwise and phenotype-driven therapeutic approach can be proposed (Table 2). In FD, treatment selection should be guided not only by disease severity, but also by the predominant clinical-inflammatory phenotype and chronicity. In classic neutrophilic-predominant FD, characterized by active pustulation and perifollicular inflammation, systemic antibiotics remain the preferred first-line therapy, particularly tetracyclines, given their combined anti-inflammatory and antimicrobial effects and their ability to control perifollicular inflammation [14]. Their relatively favorable safety profile also supports their use as initial therapy. However, their role is mainly in short- to medium-term disease control rather than durable remission. In patients with more severe inflammatory activity or refractory disease, the clindamycin–rifampicin combination may be used, particularly when *S. aureus* colonization and biofilm-driven inflammation are suspected; however, its benefits are often limited by frequent relapse after discontinuation and by safety concerns related to prolonged antibiotic exposure, including gastrointestinal intolerance, hepatotoxicity, Clostridioides difficile infection risk, drug interactions, and antimicrobial resistance [13] (Table 2). When disease persists or relapses, isotretinoin represents the most effective second-line systemic option, showing higher rates of sustained remission compared with antibiotic-based strategies, although relapse after discontinuation remains possible [17]. Its use may be particularly relevant in patients with chronic relapsing disease or clinical features suggestive of follicular occlusion. Nevertheless, monitoring for mucocutaneous adverse effects, lipid abnormalities, hepatotoxicity, and teratogenicity is required. Among second-line options, dapsone should also be considered in patients with persistent neutrophilic inflammatory activity, given its anti-neutrophilic effects and moderate rates of stable remission reported in comparative studies [11]. Importantly, patients within the FD–lichen planopilaris phenotypic spectrum or those presenting with clinical features suggestive of mixed inflammatory activity, such as prominent perifollicular erythema, scaling, or persistent inflammatory progression despite adequate antibiotic therapy, may benefit from earlier introduction of immunomodulatory therapies, such as hydroxychloroquine or short-term cyclosporine [21]. Cyclosporine should mainly be regarded as a rescue therapy for rapidly progressive inflammatory disease rather than a maintenance strategy, given the frequent relapse observed after discontinuation and its unfavorable long-term safety profile (Table 2). In highly refractory or severe cases, escalation to biologic therapies, particularly anti-TNF agents, appears justified [35]. Other targeted therapies, including IL-17/23 inhibitors, JAK inhibitors, and apremilast, may be considered in selected refractory patients, although current evidence remains limited and long-term safety data are lacking (Table 2). Importantly, adjunctive therapies may provide additional benefit in refractory FD. PDT has shown the most consistent results among non-systemic options, with reports of sustained disease control in some patients [26]. Laser-based treatments may help reduce follicular inflammation and bacterial load, while PRP has shown preliminary symptomatic improvement in small case series [28]. In DCS, the therapeutic hierarchy differs substantially and should be guided mainly by the extent of follicular occlusion, suppuration, inflammatory burden, and structural damage (Table 2). While antibiotics can be useful in early inflammatory stages, particularly to control secondary bacterial colonization, suppuration, and acute symptoms, their effects are typically transient and insufficient in chronic disease. Isotretinoin represents the preferred first-line systemic therapy in most patients, as it directly targets follicular occlusion and is associated with high response rates [42]. Maintenance or prolonged regimens may be required due to relapse risk. For patients with moderate-to-severe inflammatory disease, particularly those with overlap with HS, biologic therapies, especially anti-TNF agents, represent the next therapeutic step, with growing evidence supporting their efficacy [44]. Emerging options such as IL-17/23 inhibitors, JAK inhibitors, and apremilast may be considered in selected refractory cases, although evidence remains preliminary. Finally, surgery plays a key role in advanced fibrotic DCS with established sinus tracts and irreversible structural damage, where medical therapy alone is often insufficient [7]. A combined medical–surgical approach is frequently necessary to achieve optimal long-term disease control (Table 2). Despite the growing body of evidence, several limitations must be acknowledged. Most available data are derived from retrospective studies, case series, and case reports, with a lack of randomized controlled trials. This significantly limits the ability to establish standardized treatment algorithms or directly compare therapeutic efficacy. In addition, outcome measures are often heterogeneous, making cross-study comparisons difficult. Another important limitation is the lack of validated disease severity scores for FD and DCS, which further complicates the assessment of treatment response. Moreover, variability in treatment regimens, follow-up duration, and reporting of clinical outcomes further reduces the comparability and generalizability of findings. Small sample sizes and potential selection bias also represent relevant constraints. Finally, the absence of long-term data limits the ability to assess sustained efficacy and safety of both conventional and emerging therapies.

## 5. Conclusions

This narrative review provides an overview of current systemic therapies for FD and DCS, highlighting both established treatments and emerging targeted approaches. Accurate and timely diagnosis remains essential to guide appropriate therapeutic decisions and prevent disease progression. In this context, improved disease characterization, also supported by non-invasive diagnostic tools, may enable a more personalized approach, allowing treatment to be tailored and adjusted according to clinical course. Differences in underlying pathogenic mechanisms likely explain the variability in treatment response and support a mechanism-based management strategy. When topical therapy is insufficient, systemic treatment is required, with combined approaches often providing better disease control. Given the chronic and relapsing nature of these conditions, long-term strategies are frequently necessary, and a multidisciplinary approach may further optimize outcomes. Future studies with larger cohorts are needed to better clarify disease pathogenesis, standardize outcome measures, and support the development of targeted therapies aimed at improving patients’ quality of life.

## Figures and Tables

**Table 1:** Differential diagnosis of scarring alopecias.

Primary Cicatricial Alopecias (PCA)	Folliculitis Decalvans (FD)	Dissecting Cellulitis of the Scalp (DCS)	Lichen Planopilaris (LPP)	Discoid Lupus Erythematosus (DLE)	Central Centrifugal Cicatricial Alopecia (CCCA)	Acne Keloidalis Nuchae (AKN)
**Type of PCA**	Neutrophilic/mixed	Neutrophilic/mixed	Lymphocytic	Lymphocytic	Lymphocytic	Mixed (neutrophilic → fibrosing)
**Pathogenesis (main driver)**	Microbial dysbiosis + neutrophilic inflammation	Follicular occlusion + deep inflammation	Autoimmune (T-cell mediated)	Autoimmune (interface dermatitis)	Multifactorial	Follicular irritation + occlusion + aberrant wound healing
**Typical clinical presentation**	Tufted hairs, pustules, crusts, patchy scarring alopecia	Painful nodules, abscesses, sinus tracts, suppuration	Patchy alopecia with perifollicular erythema and scaling	Erythematous plaques with scaling, dyspigmentation, atrophy	Progressive vertex alopecia, centrifugal	Firm papules/plaques on nape, keloid-like scars, scarring alopecia
**Pustules/suppuration**	Common	Prominent	Rare	Rare	Absent	Early lesions may have pustules
**Distribution**	Vertex/occiput	Vertex, occiput	Multifocal scalp	Photo-exposed scalp	Vertex	Occipital scalp/nape of neck
**Trichoscopy**	Hair tufting, scaling, crusts	Yellow areas, pustules, sinus openings	Perifollicular scaling and erythema	Follicular plugging, telangiectasia	Peripilar white/gray halos	Perifollicular papules, white areas (fibrosis), hair loss
**Associated conditions**	*S. aureus* colonization	Hidradenitis suppurativa	Lichen planus	Systemic lupus	Hair practices	Mechanical irritation (shaving), curly hair phenotype

Overview of the differential diagnosis among the major primary scarring alopecias based on in-flammatory profile, pathogenesis, clinical manifestations, trichoscopy, and associated conditions. [4,5,6,7,9,20,54,55,56,57,58,59].

**Table 2:** Systemic and adjunctive therapies in FD and DCS.

Therapy	Mechanism of Action	Indications in FD	Indications in DCS	Clinical Considerations
**Tetracyclines**	Antimicrobial + anti-inflammatory (↓ neutrophil chemotaxis, MMP inhibition)	First-line in mild–moderate disease	Early inflammatory stages	Partial responses; limited durability
**Clindamycin + Rifampicin**	Antimicrobial + anti-inflammatory (biofilm penetration)	Moderate–severe or refractory disease	Active inflammatory disease with suppuration	High initial response; frequent relapse
**Other antibiotics (macrolides, TMP-SMX)**	Antimicrobial and anti-inflammatory	Alternative options	Alternative options	Limited evidence; minimal long-term impact
**Isotretinoin**	Normalizes follicular keratinization, anti-inflammatory	Second-line and adjunctive	First-line systemic in moderate–severe disease	High response in DCS; relapse possible
**Hydroxychloroquine**	Immunomodulatory	Adjunctive	Limited role	Selected cases
**Cyclosporine**	T-cell inhibition	Refractory FD	Limited role	Short-term use due to safety
**Dapsone**	Anti-neutrophilic	Second-line and adjunctive	Limited role	Requires monitoring
**Photodynamic therapy (PDT)**	Antimicrobial + anti-inflammatory	Adjunctive	Limited role	Heterogeneous evidence
**Laser therapies**	↓ follicular inflammation, bacterial load	Adjunctive	Limited role	Limited evidence
**Platelet-rich plasma (PRP)**	Tissue modulation, anti-inflammatory	Adjunctive	Limited role	Very limited data
**TNF-α inhibitors**	Block TNF-mediated inflammation	Refractory FD	Refractory DCS; HS overlap	Most robust biologic evidence
**IL-17 inhibitors**	Inhibit Th17-mediated inflammation	Refractory FD	Refractory DCS; HS overlap	Limited but promising evidence
**IL-23 inhibitors**	Downstream Th17 modulation	Refractory FD	Refractory DCS	Case reports only
**JAK inhibitors**	Broad cytokine signaling inhibition	Refractory FD	Refractory DCS	Limited but promising evidence
**PDE-4 inhibitors (apremilast)**	↓ pro-inflammatory cytokines via cAMP	Refractory FD	Refractory DCS	Limited evidence
**Surgery**	Removal of diseased tissue	Not indicated	Advanced disease with sinus tracts	Combine with systemic therapy

↓ stands for “decreasing”. Summary of systemic and adjunctive therapies used in folliculitis decalvans and dissecting cellulitis of the scalp, including mechanisms of action, clinical indications, and therapeutic considerations [5,6,10,12,13,15,16,20,21,22,23,25,27,28,34,35,36,37,38,39,40,41,43,45,46,47,48,49,50,51,52,53].

## Data Availability

No new data were created or analyzed in this study. Data sharing is not applicable to this article.

## Abbreviations

The following abbreviations are used in this manuscript:

PCA	Primary Cicatricial Alopecias
FD	Folliculitis Decalvans
DCS	Dissecting Cellulitis of the Scalp
HS	Hidradenitis Suppurativa
LPP	Lichen Planopilaris
DLE	Discoid Lupus Erythematosus
CCCA	Central Centrifugal Cicatricial Alopecia
AKN	Acne Keloidalis Nuchae
NAHRS	North American Hair Research Society
EADV	European Academy of Dermatology and Venereology
Nd:YAG	Neodymium-doped Yttrium Aluminum Garnet
PDT	Photodynamic Therapy

## References

[1] Villablanca S., Fischer C., García-García S.C., Mascaró-Galy J.M., Ferrando J. (2017). Primary Scarring Alopecia: Clinical-Pathological Review of 72 Cases and Review of the Literature. Ski. Appendage Disord..

[2] Whiting D.A. (2001). Cicatricial alopecia: Clinico-pathological findings and treatment. Clin. Dermatol..

[3] Olsen E.A., Bergfeld W.F., Cotsarelis G., Price V.H., Shapiro J., Sinclair R., Solomon A., Sperling L., Stenn K., Whiting D.A. (2003). Summary of North American Hair Research Society (NAHRS)-sponsored Workshop on Cicatricial Alopecia, Duke University Medical Center, February 10 and 11, 2001. J. Am. Acad. Dermatol..

[4] Ezemma O., Devjani S., Kelley K.J., Senna M.M. (2023). Treatment modalities for lymphocytic and neutrophilic scarring alopecia. J. Am. Acad. Dermatol..

[5] Cummins D.M., Chaudhry I.H., Harries M. (2021). Scarring Alopecias: Pathology and an Update on Digital Developments. Biomedicines.

[6] Waśkiel-Burnat A., Starace M., Iorizzo M., Katoulis A., Apalla Z., Asfour L., Freites-Martinez A., Ioannides D., Jafari S.M.S., Kelati A. (2025). Management of folliculitis decalvans: The EADV task force on hair diseases position statement. J. Eur. Acad. Dermatol. Venereol..

[7] Masson R., Jeong C.Y., Ma E., Crew A.B., Fragoso N.M., Shi V.Y., Hsiao J.L. (2023). Treatments for Dissecting Cellulitis of the Scalp: A Systematic Review and Treatment Algorithm. Dermatol. Ther..

[8] Bolduc C., Sperling L.C., Shapiro J. (2016). Primary cicatricial alopecia: Other lymphocytic primary cicatricial alopecias and neutrophilic and mixed primary cicatricial alopecias. J. Am. Acad. Dermatol..

[9] Awad A., Kho Y.C., Asfour L., Sladden M., Prakash S., Nirenberg A., Fakih A., El Sayed F., Bhoyrul B. (2026). Paediatric-Onset Folliculitis Decalvans and Lichen Planopilaris Phenotypic Spectrum: Is It a Different Disease?. Exp. Dermatol..

[10] Miteva M., Tosti A. (2013). Dermoscopy guided scalp biopsy in cicatricial alopecia. J. Eur. Acad. Dermatol. Venereol..

[11] Wyche J., Senna M., Aguh C. (2025). Approach to Scarring Alopecia. JAMA Dermatol..

[12] Farnina L., Cazzaniga S., Hunger R.E., Heidemeyer K., De Viragh P.A., Jafari S.M.S. (2025). Treatment Survival and Reasons for Discontinuation in Patients with Recalcitrant Folliculitis Decalvans. Acta Derm. Venereol..

[13] Singh G., Pathania Y., Goswami P., Tyagi S., Patel T. (2025). Folliculitis Decalvans: An Uncommon Case Report with Review of Literature. Int. J. Appl. Basic Med. Res..

[14] Moreno-Arrones O.M., del Campo R., Saceda-Corralo D., Jimenez-Cauhe J., Ponce-Alonso M., Serrano-Villar S., Jaén P., Paoli J., Vañó-Galván S. (2021). Folliculitis decalvans microbiologic signature is specific for disease clinical phenotype. J. Am. Acad. Dermatol..

[15] Kashikar Y., Saoji V., Madke B., Chandak M.S., Meghe S. (2024). Successful Management of Folliculitis Decalvans. Cureus.

[16] Vañó-Galván S., Molina-Ruiz A., Fernández-Crehuet P., Rodrigues-Barata A.R., Arias-Santiago S., Serrano-Falcón C., Martorell-Calatayud A., Barco D., Pérez B., Serrano S. (2015). Folliculitis decalvans: A multicentre review of 82 patients. J. Eur. Acad. Dermatol. Venereol..

[17] Tietze J., Heppt M., von Preußen A., Wolf U., Ruzicka T., Wolff H., Sattler E. (2015). Oral isotretinoin as the most effective treatment in folliculitis decalvans: A retrospective comparison of different treatment regimens in 28 patients. J. Eur. Acad. Dermatol. Venereol..

[18] Melo D.F., Machado C.J., Bordignon N.L., Silva L.L., Ramos P.M. (2020). Lymecycline as a treatment option for dissecting cellulitis and folliculitis decalvans. Dermatol. Ther..

[19] Jawade S., Singh S., Quazi S., Dudhe M., Neazee S., Meghe S.R., Jawade S. (2024). Effective Treatment of Folliculitis Decalvans with a Combination of Oral Isotretinoin and Rifampicin. Cureus.

[20] Aksoy B., Hapa A., Mutlu E. (2018). Isotretinoin treatment for folliculitis decalvans: A retrospective case-series study. Int. J. Dermatol..

[21] Melián-Olivera A., Moreno-Arrones Ó., Burgos-Blasco P., Hermosa-Gelbard Á., Jaén-Olasolo P., Vañó-Galván S., Saceda-Corralo D. (2024). Clinical Characterization and Treatment Response of Folliculitis Decalvans Lichen Planopilaris Phenotypic Spectrum: A Unicentre Retrospective Series of 31 Patients. Acta Derm. Venereol..

[22] Doche I., Sotto M.N., Hordinsky M.K., Melhado I.P., Gerlero P., Rivitti-Machado M.C. (2025). Hydroxychloroquine may be beneficial as an adjuvant therapy for folliculitis decalvans: A 5-year retrospective study with 49 patients. Clin. Exp. Dermatol..

[23] Jerjen R., Meah N., De Carvalho L.T., Wall D., Gunatheesan S., Sinclair R. (2021). Effective treatment of folliculitis decalvans with cyclosporin: A case series. Australas. J. Dermatol..

[24] Melián-Olivera A., Burgos-Blasco P., Selda-Enríquez G., Suárez-Valle A., Miguel-Gómez L., Vañó-Galván S., Saceda-Corralo D. (2022). Topical dapsone for folliculitis decalvans: A retrospective cohort study. J. Am. Acad. Dermatol..

[25] Rózsa P., Varga E., Gyulai R., Kemény L. (2024). Carbon-dioxide laser-associated PDT treatment of folliculitis decalvans. Int. J. Dermatol..

[26] Yang L., Chen J., Tong X., Gao L., Ding S., Guo A. (2021). Photodynamic therapy should be considered for the treatment of folliculitis decalvans. Photodiagnosis Photodyn. Ther..

[27] Collier N.J., Allan D., Pesantes F.D., Sheridan L., Allan E. (2018). Systemic photodynamic therapy in folliculitis decalvans. Clin. Exp. Dermatol..

[28] Parlette E.C., Kroeger N., Ross E.V. (2004). Nd:YAG laser treatment of recalcitrant folliculitis decalvans. Dermatol. Surg..

[29] Suh S., Nguyen C., Zhao L., Mesinkovska N.A. (2021). The role of platelet-rich plasma therapy in refractory folliculitis decalvans. JAAD Case Rep..

[30] Fakhoury T. (2024). Recalcitrant Folliculitis Decalvans Treatment Outcomes with Biologics and Small Molecule Inhibitors. Cutis.

[31] Alsantali A., Baghdadi R., Alghamdi Y., Abbas E.B., Ghazi R. (2023). Folliculitis decalvans managed with adalimumab: A case report. Clin. Case Rep..

[32] Dupont A., Eyraud A., Milpied B., De Bataille S., Cassassa E., Darrigade A.-S., Barnetche T., Doutre M.-S., Matard B., Beylot-Barry M. (2023). Efficacy and Safety of Tumour Necrosis Factor-α Antagonists for Folliculitis Decalvans: A Retrospective Case-series Pilot Study. Acta Derm. Venereol..

[33] Lobato-Berezo A., Pujol R.M. (2022). Refractory folliculitis decalvans treated with biologics: Case series in 4 situations. Dermatol. Ther..

[34] Hoy M., Böhm M. (2022). Therapy-refractory folliculitis decalvans treated with certolizumab pegol. Int. J. Dermatol..

[35] Iorizzo M., Starace M., Vano-Galvan S., Piraccini B.M., Reygagne P., Rudnicka L., Silyuk T., Sinclair R., Tosti A. (2022). Refractory folliculitis decalvans treated with adalimumab: A case series of 23 patients. J. Am. Acad. Dermatol..

[36] Franciozi A.B., Sotto M.N., Rivitti-Machado M.C.M., Pagliari C., Doche I. (2025). Evidence of interleukin-17-secreting mast cells in scalp lesions of folliculitis decalvans points to new therapeutic targets in recalcitrant lesions. Clin. Exp. Dermatol..

[37] Ismail F.F., Sinclair R. (2020). Successful treatment of refractory folliculitis decalvans with secukinumab. Australas. J. Dermatol..

[38] Moussa A., Asfour L., Eisman S., Bhoyrul B., Sinclair R. (2022). Successful treatment of folliculitis decalvans with baricitinib: A case series. Australas. J. Dermatol..

[39] Lasheras-Pérez M.A., Garrido-Ruiz M.C., Lobato-Berezo A., Palacios-Diaz R.D., Albert-Cobo I., Gallo G., Velasco-Tamariz V., Ruiz-Villaverde R., Gil-Redondo R., Pujol-Marco C. (2025). Immunohistochemical Characterization of JAK Family Expression in the Primary Inflammatory Infiltrate of Lichen Planopilaris and Folliculitis Decalvans, With an Exploratory Clinical Assessment of Oral JAK Inhibitors. Int. J. Dermatol..

[40] Dethier C., Azirar S., Verluyten L., Boonen H., Grosber M., Gutermuth J. (2025). Response to Fässler et al’s “Successful treatment of refractory folliculitis decalvans with apremilast”. JAAD Case Rep..

[41] de Viragh P.A., Jafari S.M.S. (2025). Response to Dethier et al, “Experience in nine additional cases of apremilast for therapy-resistant folliculitis decalvans”. JAAD Case Rep..

[42] Guo W., Zhu C., Stevens G., Silverstein D. (2021). Analyzing the Efficacy of Isotretinoin in Treating Dissecting Cellulitis: A Literature Review and Meta-Analysis. Drugs R&D.

[43] Chu S., Michelle L., Ekelem C., Sung C.T., Rojek N., Mesinkovska N.A. (2021). Oral isotretinoin for the treatment of dermatologic conditions other than acne: A systematic review and discussion of future directions. Arch. Dermatol. Res..

[44] Sanchez-Diaz M., Martinez-Lopez A., Salvador-Rodriguez L., Montero-Vilchez T., Arias-Santiago S., Molina-Leyva A. (2021). The role of biologic treatment in special scenarios in hidradenitis suppurativa: Facial and nape phenotype, dissecting cellulitis of the scalp, and lymphedema. Dermatol. Ther..

[45] Ramos F.J.M., Berna-Rico E., Suarez-Valle A., Puchades A.M., Vañó-Galván S., García-Ruiz R., Corralo D.S. (2024). Determinant factors of biological therapy for dissecting cellulitis of the scalp. JDDG J. Dtsch. Dermatol. Ges..

[46] Alzahrani M., Coste V., Konstantinou M.P., Reguiai Z., Villani A., Hotz C., Viguier M., Pruvost-Balland C., Dupuy A., Wolkenstein P. (2023). Treatment of dissecting cellulitis of the scalp with tumour necrosis factor inhibitors: A retrospective multicentre study. Clin. Exp. Dermatol..

[47] Sabat R., Alavi A., Wolk K., Wortsman X., McGrath B., Garg A., Szepietowski J.C. (2025). Hidradenitis suppurativa. Lancet.

[48] Awad A., Sinclair R. (2022). Treatment of dissecting cellulitis of the scalp with Tildrakizumab. Australas. J. Dermatol..

[49] Heidari N., Pour R.G.K., Farshbafnadi M., Heidari A., Ghane Y. (2025). A systematic review of tumor necrosis factor-α blockers, anti-interleukins, and small molecule inhibitors for dissecting cellulitis of the scalp treatment. Orphanet J. Rare Dis..

[50] Al-Mamoori A., Al-Badri S.G.A., Sayed S.B.H., Al-Daraji W. (2025). Janus kinase inhibitors as a breakthrough treatment for refractory dissecting cellulitis of the scalp. Eur. J. Case Rep. Intern. Med..

[51] Islam Z., Toker M., Gandhi I.M., Sher A., Campton K. (2024). Improvement of Recalcitrant Dissecting Cellulitis of the Scalp After a Trial of Upadacitinib. Cureus.

[52] Qiu M., Man X., Jing J. (2025). Dissecting Cellulitis of the Scalp Successfully Treated with a Combination of Ixekizumab and Tofacitinib. J. Inflamm. Res..

[53] Baneu N.-S., Bloancă V.A., Szilagyi D., Cristodor P., Pesecan A., Bratu T.I., Crăiniceanu Z.P. (2021). Surgical management of dissecting cellulitis of the scalp using free latissimus dorsi flap and meshed split-thickness skin graft: A case report. Medicine.

[54] Spiers J., Vinnicombe Z., Singh G., Pouncey A., McEvoy H. (2021). Severe dissecting scalp cellulitis successfully treated with serial excisions in combination with anti-TNF therapy. Ann. R. Coll. Surg. Engl..

[55] Valtellini L., Perego G., Aromolo I.F., Moltrasio C., Maronese C.A., Marzano A.V., Sechi A., Avallone G. (2026). Dissecting Cellulitis of the Scalp: Current Insights and Therapeutic Advances. Am. J. Clin. Dermatol..

[56] Lawson C.N., Bakayoko A., Callender V.D. (2021). Central Centrifugal Cicatricial Alopecia: Challenges and Treatments. Dermatol. Clin..

[57] Alves J.P., Heck R. (2022). Acne Keloidalis Nuchae. J. Cutan. Med. Surg..

[58] Katoulis A.C., Pappa G., Sgouros D., Markou E., Kanelleas A., Bozi E., Ioannides D., Rudnicka L. (2025). A Three-Step Diagnostic Algorithm for Alopecia: Pattern Analysis in Trichoscopy. J. Clin. Med..

[59] Otberg N., Kang H., Alzolibani A.A., Shapiro J. (2008). Folliculitis decalvans. Dermatol. Ther..

[60] Uchiyama M., Harada K., Tobita R., Irisawa R., Tsuboi R. (2021). Histopathologic and dermoscopic features of 42 cases of folliculitis decalvans: A case series. J. Am. Acad. Dermatol..

[61] Lee C.-N., Chen W., Hsu C.-K., Weng T.-T., Lee J.Y.-Y., Yang C.-C. (2018). Dissecting folliculitis (dissecting cellulitis) of the scalp: A 66-patient case series and proposal of classification. JDDG J. Dtsch. Dermatol. Ges..

